# Transcriptomics of Acute DENV-Specific CD8+ T Cells Does Not Support Qualitative Differences as Drivers of Disease Severity

**DOI:** 10.3390/vaccines10040612

**Published:** 2022-04-14

**Authors:** Alba Grifoni, Hannah Voic, Esther Dawen Yu, Jose Mateus, Kai Mei Yan Fung, Alice Wang, Grégory Seumois, Aruna D. De Silva, Rashika Tennekon, Sunil Premawansa, Gayani Premawansa, Rashmi Tippalagama, Ananda Wijewickrama, Ashu Chawla, Jason Greenbaum, Bjoern Peters, Vijayanand Pandurangan, Daniela Weiskopf, Alessandro Sette

**Affiliations:** 1 La Jolla Institute for Immunology (LJI), La Jolla, CA 92037, USA; agrifoni@lji.org (A.G.); hannah.voic12@ncf.edu (H.V.); dyu@lji.org (E.D.Y.); jmtrivino@lji.org (J.M.); kfung@lji.org (K.M.Y.F.); awang@lji.org (A.W.); gregory@lji.org (G.S.); dslv90@yahoo.com (A.D.D.S.); rtippalagama@lji.org (R.T.); ashu@lji.org (A.C.); jgbaum@lji.org (J.G.); bpeters@lji.org (B.P.); vijay@lji.org (V.P.); dweiskopf@lji.org (D.W.); 2 Department of Paraclinical Sciences, Faculty of Medicine, General Sir John Kotelawala Defense University, Ratmalana 10390, Sri Lanka; rashika29@gmail.com; 3 Department of Zoology and Environment Sciences, University of Colombo, Colombo 00300, Sri Lanka; suviprema@gmail.com; 4 North Colombo Teaching Hospital, Ragama 11010, Sri Lanka; gavisprema@gmail.com; 5 National Institute of Infectious Diseases, Gothatuwa, Angoda 10620, Sri Lanka; anandawijewickrama@hotmail.com; 6 Department of Medicine, Division of Infectious Diseases and Global Public Health, University of California, La Jolla, CA 92037, USA

**Keywords:** CD8+ T cells, DENV, hemorrhagic disease, transcriptome, phenotypes

## Abstract

While several lines of evidence suggest a protective role of T cells against disease associated with Dengue virus (DENV) infection, their potential contribution to immunopathology in the acute phase of DENV infection remains controversial, and it has been hypothesized that the more severe form of the disease (dengue hemorrhagic fever, DHF) is associated with altered T cell responses. To address this question, we determined the transcriptomic profiles of DENV-specific CD8+ T cells in a cohort of 40 hospitalized dengue patients with either a milder form of the disease (dengue fever, DF) or a more severe disease form (dengue hemorrhagic fever, DHF). We found multiple transcriptomic signatures, one associated with DENV-specific interferon-gamma responding cells and two other gene signatures, one specifically associated with the acute phase and the other with the early convalescent phase. Additionally, we found no differences in quantity and quality of DENV-specific CD8+ T cells based on disease severity. Taken together with previous findings that did not detect altered DENV-specific CD4 T cell responses, the current analysis argues against alteration in DENV-specific T cell responses as being a correlate of immunopathology.

## 1. Introduction

Dengue infection and disease have increased more than eight-fold over the last two decades, reaching 5.2 million infections and 4032 deaths (reported on 19 May 2021; www.WHO.org (accessed on 16 March 2022)). Dengue disease is caused by infection with the Dengue virus (DENV), a mosquito-borne flavivirus. DENV is endemic in more than 100 countries, mainly localized in the tropical and sub-tropical geographical areas, and is associated with the potential for even greater spread as a consequence of climate change and global warming [1,2]. Upon infection, the average incubation period is 4–7 days and followed by a spectrum of disease manifestations ranging from mild febrile symptoms (dengue fever, DF) to the more severe clinical symptomatology, characterized by hemorrhagic fever (DHF) with plasma leakage, and in the most severe cases hypovolemic shock that may be followed by multi-organ failure (dengue shock syndrome, DSS) [3]. A single dengue vaccine, CYD-TDV, is currently licensed and indicated solely for subjects previously exposed to one or multiple DENV serotypes [3,4], thus underlining a need for further improvement of dengue vaccines. CYD-TDV is based on the delivery of the envelope (Env) protein derived from DENV1-4 serotypes by a yellow fever (YF) backbone as a vector [5]. Since Env is a strong target for antibody responses, but a weak target for cellular immunity [6,7,8], vaccine performance might be enhanced by the inclusion of other DENV antigens that are a dominant target of T cell responses, for example, by the use of DENV attenuated viruses as vaccines [5,9,10]. In this context, a detailed understanding of the relative role of humoral and cellular immunity in disease protection and immunopathology is key for the design of improved vaccine concepts. Several studies have shown that antibody response can exacerbate disease severity through the phenomenon of antibody-dependent enhancement (ADE), where antibodies generated after primary infection can facilitate viral entry and replication in target cells and potentially trigger a cytokine storm potentially responsible for the leakage observed in severe dengue cases [11,12,13,14]. In the case of T cells, it was hypothesized that suboptimal cross-reactive T cells might be unable to control heterotypic infection and rather be associated with an altered phenotype and severe disease [15]. This original hypothesis was not confirmed by subsequent studies [16,17,18]. In contrast, several independent studies in the past years have consistently shown a potential contribution of T cells in controlling DENV infection in mice and humans [17,19,20,21,22]. In humans, we and others have shown that in acute infection, DENV-specific CD4+ T cells are not characterized by changes in phenotype or functional attributes, arguing against the notion that altered CD4+ T-cell phenotype or function may be a determinant of severe dengue disease [16,17,23]. However, the role of CD8+ T cells in acute infection and modulation of disease severity remains controversial. To address this point, we compared the transcriptomic profiles of DENV-specific CD8+ T cells in a cohort of dengue hospitalized donors with either mild (DF) or moderate (DHF) forms of dengue disease and compared the quantity as well as the quality of CD8+ T cell responses.

## 2. Materials and Methods

### 2.1. Human Blood Samples

Human blood samples were collected in the North Colombo Teaching Hospital, Ragama in Gampaha District, Sri Lanka, and in the National Institute of Infectious Diseases, Gothatuwa, Angoda, Sri Lanka. The current study was constrained by the availability of samples collected in the hospital settings. As such, a formal sample size calculation was not performed before study initiation. PBMC were isolated from small blood volumes from anonymous patients at the time of diagnosis/admission and at discharge from the hospital or after recovery from the disease; each patient provided informed consent and was assigned a study identification number with clinical information recorded. According to the 1997 WHO classification of dengue disease [24], patients were diagnosed with dengue fever (DF) or dengue hemorrhagic fever (DHF) with the presence of fever, thrombocytopenia, hemorrhagic manifestations, and plasma leakage. In the current study, all DHF patients recruited had ultrasound/sonogram-confirmed fluid accumulation as evidence of plasma leakage. The diagnosis of DENV infection was also confirmed if at least one of the following criteria was met in the acute phase serum: (1) positive reverse transcription-polymerase chain reaction (RT-PCR) of DENV RNA, (2) positive serology for dengue IgM, or (3) positive dengue-specific non-structural antigen-1 (NS1). One limitation of this cohort enrollment is the low rate of positive PCR samples that did not allow us to analyze this cohort based on the infected DENV serotype. To overcome this issue, the samples were collected in the 2014–2016 range, and an even number of samples were collected in the DF and DHF cohort. The sample collection was consistent with a prevalence of DENV1 serotype [25].

### 2.2. Serology

The IgG serology was additionally carried out to distinguish primary versus secondary DENV infections. Accordingly, we classified the donors as primary infection if seropositive for IgM but not IgG, and secondary infection for all donors seropositive for IgG disregarding the PCR and IgM status. Seventy and ninety percent of DF and DHF cohorts, respectively, showed seropositivity to IgG, suggesting that the majority of the cohort is characterized by secondary or multiple DENV infections. The serology was performed by DENV-specific IgG ELISAs and flow cytometry-based neutralization assays, as previously described [26]. One limitation of this study is the potential cross-reactivity with JEV, reported previously in Sri Lanka [27]. Since then, a vaccine campaign for children has been put in place using a more effective live-attenuated WHO-approved vaccination that has notably reduced acute infections minimizing the issues of misclassifications.

### 2.3. Peptides and Megapools

To stimulate DENV-specific CD8+ T cells, 268 DENV-specific CD8 epitopes that account for 90% of the IFNγ response in both Sri Lankan and Nicaraguan cohorts [6,28,29] were synthesized (A&A, San Diego, CA, USA), resuspended in DMSO, pooled, lyophilized, and resuspended, as previously reported [30] at 1 mg/mL.

### 2.4. IFN-γ Capture Assay and Cell Sorting for RNA-Seq

**Figure 1:**
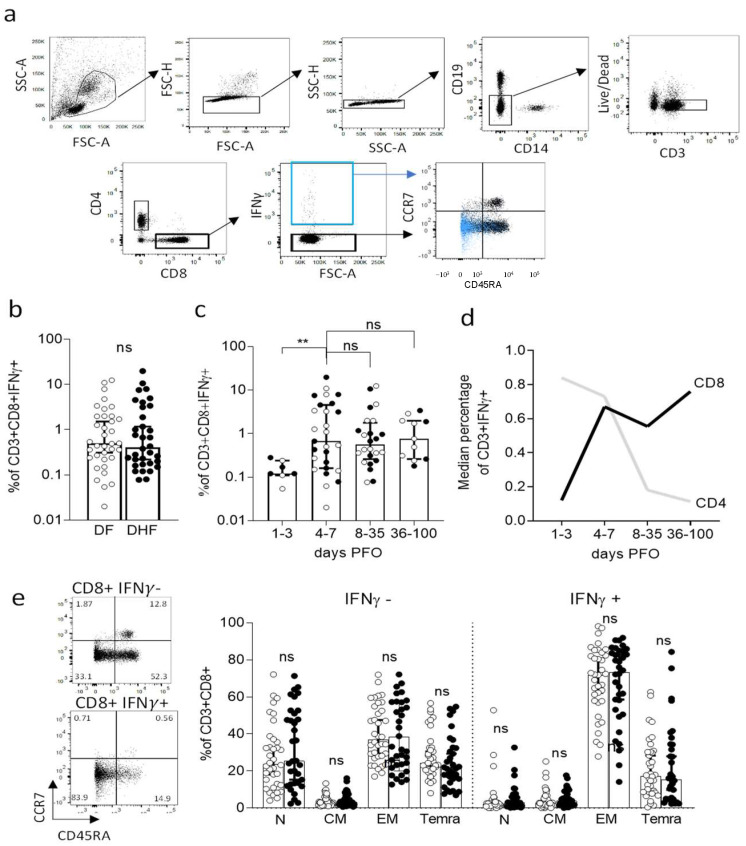
Flow cytometry analysis of DENV-specific CD8+ T cells in DENV hospitalized donors. (**a**) Gating strategy. (**b**) Magnitude of CD3 + CD8+ IFNγ+ T cells responses as a function of disease severity. (**c**) Magnitude of CD3 + CD8+ IFNγ+ T cells responses as a function of days post fever onset (PFO). (**d**) Median percentage of CD3 + CD8+ IFNγ+ T cells compared to previous data generated on CD3 + CD4 + IFNγ+ T cells (3). (**e**) Memory phenotype of CD3 + CD8+ T cells in the IFNγ− and IFNγ+ compartments as a function of disease severity. In white, DF donors, in black DHF. ** *p* < 0.01; ns = not significant. Statistical comparisons performed with Mann–Whitney test. N = naïve; CM = central memory; EM = effector memory; Temra = terminal differentiated effector memory cells.

Human PBMC were thawed and rested overnight at 37 °C in HR5 culture media consisting of RPMI (catalog #RP-21, Omega Scientific, Inc., Tarzana, CA, USA), Human serum at 5% (catalog #100-512, Gemini Bio-Products, Sacramento, CA, USA), 2 mM l-alanyl-l-glutamine (GlutaMAX-I, catalog #35050061, Thermo Fisher Scientific, Waltham, MA, USA ), 100 U/mL penicillin, and B 100 μg/mL streptomycin (catalog# 400-109, Gemini Bio-Products, Sacramento, CA, USA). After overnight resting, cells were stimulated with DENV CD8 megapool (1 ug/mL final concentration) for 3 h at 37 °C. IFNγ producing cells were labeled using an IFN-γ Secretion Assay–Detection kit (PE) (catalog #130-054-202, Miltenyi Biotec, Bergisch Gladbach, North Rhine-Westphalia, Germany) according to manufacturer’s instructions. Cells were additionally stained with anti-CD3 (AF700, clone OKT3, catalog #317340 Biolegend, San Diego, CA, USA), CD4 (APC eFluor 780, clone RPA-T4 catalog #301042, eBioscience, San Diego, CA, USA) CD8 (BV650, clone RPA-T8 catalog #47-0049-42, Biolegend, San Diego, CA, USA), CD14 (V500, clone M5E2, catalog# 561391, BD Biosciences, San Diego, CA, USA), CD19 (V500, clone HIB19, catalog #561121, BD Biosciences, San Diego, CA, USA), CD45RA (eFluor 450, clone HI100, catalog #48-0458-42, eBioscience, San Diego, CA, USA), CCR7 (PerCP Cy5.5, clone G043H7, catalog #353220, Biolegend, San Diego, CA, USA), and viability dye (ef506, catalog #65-0866-18, eBioscience, San Diego, CA, USA). The CD8+ T cell population was isolated according to the gating strategy detailed in Figure 1a. Two hundred cells each from CD8+ IFNγ+ and CD8+ IFNγ− cell populations were sorted using a FACSAria cell sorter (BD Biosciences, San Diego, CA, USA). Data were analyzed using Flowjo version 10.6 (Treestar Inc., Ashland, OR, USA). Statistical analysis and visualization were performed using Prism8.4.3 (GraphPad, San Diego, CA, USA).

### 2.5. Microscaled RNA-Sequencing Assay

Sorted cells (200 to 400) were directly collected in 0.2 mL PCR tubes (Axygen, Union city, CA, USA) containing 8 µL of lysis buffer (0.2% Triton X-100 [catalog #X100-100 mL, Sigma-Aldrich, St Louis, MO, USA], 0.2 mM dNTP [catalog #R0193, ThermoFisher Scientific Waltham, MA, USA], 1 U/uL recombinant RNAse Inhibitor [catalog #2313A, Takara Bio, Kusatsu, Shiga, Japan]). Once cells were collected, tubes were vortexed, spun down, and stored at −80 °C. Microscaled RNA-Seq was performed according to the Smart-Seq2 protocol, as previously reported [31,32]. Briefly, mRNA was captured using poly-dT oligos and directly reverse-transcribed into full-length cDNA using the template-switching oligo. cDNA was amplified by PCR for 19 cycles and purified using the AMPure XP magnetic bead (0.9:1 *v*/*v* ratio, Beckman Coulter, Brea, CA, USA); 1 ng of cDNA from each sample was used to prepare dual-index barcoded standard NextEra XT Library (NextEra XT DNA library prep kit and index kits, Illumina, San Diego, CA, USA). Both whole transcriptome amplification and sequencing library preparations were performed in a 96-well format to reduce assay-to-assay variability. Quality control steps were included to determine the optimal number of PCR preamplification cycles and library fragment size. Samples that failed quality controls were eliminated from downstream steps. Libraries that passed quality controls were pooled at equimolar concentration, loaded, and sequenced on the HiSeq 2500 (Illumina, San Diego, CA, USA). Libraries were sequenced to obtain more than 8 million 50-bp single-end reads (HiSeq Rapid Run Cluster and SBS Kit v2, Illumina, San Diego, CA, USA) mapping uniquely to mRNA reference, generating a total of about 204.3 million mapped reads (median of about 8.6 million filtered mapped reads per sample) libraries.

### 2.6. RNA-Seq Analysis

The paired-end reads that passed Illumina filters were filtered for reads aligning to tRNA, rRNA, adapter sequences, and spike-in controls. The reads were then aligned to the GRCh38 reference genome and Gencode v27 annotations using STAR (v2.6.1, Stamford, CT, USA) (52). DUST scores were calculated with PRINSEQ lite v0.20.3 [33], and low-complexity reads (DUST > 4) were removed from the BAM files. The alignment results were parsed via SAMtools [34] to generate SAM files. Read counts to each genomic feature were obtained with the featureCounts (v 1.6.5) [35] using the default option along with a minimum quality cut-off (Phred > 10). After removal of absent features (zero counts in all samples), the raw counts were then imported to DESeq2 v1.28.1 to identify the differentially expressed genes between groups [36]. *p*-values for differential expression are calculated using the Wald test for differences between the base means of two conditions. These *p*-values are then adjusted for multiple test correction using the Benjamini Hochberg algorithm [37]. Genes with an adjusted *p*-value ≤ 0.05 and an absolute shrunken log2 fold change > 1.5 were considered differentially expressed between CD8+ IFNγ+ and CD8+ IFNγ− groups. Genes with an adjusted *p*-value ≤ 0.05 and an absolute shrunken log2 fold change > 1 were considered differentially expressed between CD8+ IFNγ+ 4–7 days from fever onset and CD8+ IFNγ+ 8+ days from fever onset groups. Principal component analysis (PCA) was performed using the ‘prcomp’ function in R. The sequences used in this article have been submitted to the Gene Expression Omnibus under accession number GSE174482 (http://www.ncbi.nlm.nih.gov/geo/ (accessed on 21 September 2021)).

### 2.7. WGCNA Analysis

Weighted gene co-expression network analysis (R package WGCNA v1.69) was performed for genes with median DESeq2 normalized counts greater than 10 in either of the 2 groups IFNγ− or IFNγ+ [38,39], with an adjusted *p*-value ≤ 0.05 and an absolute foldchange cut-off of 1.5 in the DE analysis between IFNγ+ and IFNγ− samples. A total of 5 co-expression modules across 992 genes were identified; the genes that were not assigned to any of these modules were kept in a separate gray module. Each gene was univocally assigned to a single module. The genes that were assigned to a module but had a negative module membership value (genes with low intramodular connectivity) were also assigned to the gray module. The gene expression profile of a module was summarized by module eigengene, which is defined as the principal component of the module. *t*-tests were performed to compare module eigengene values between samples from each of the two given groups, and all of the modules were found to have significantly different gene expression profiles between the two groups. A row-scaled heatmap of log-transformed normalized counts of these DE genes was created with the ‘heatmap.2′ function in the ggplots library in R v4.0.3. The modules were used as RowSideColors. The gene function was inferred by using Metascape [40].

## 3. Results

### 3.1. Selection of a Dengue Hospitalized Cohort to Investigate the Role of CD8+ T Cells

A total of 40 dengue hospitalized patients were recruited to the study for the purpose of analyzing the transcriptomic profiles associated with DENV-specific CD8+ T cells as a function of dengue disease severity (Table 1).

Of those, twenty subjects were associated with dengue hemorrhagic fever (DHF) with sonogram-confirmed plasma leakage, and twenty were associated with severe dengue fever (DF) with no signs of plasma leakage, according to the international WHO criteria 1997 [24] and described in more details in the methods. The two DHF and DF cohorts were recruited in the 2014–2016 time period and were generally matched in terms of general demographic features (Table 1). The median age of the DF cohort was 33 versus 30 of the DHF cohort, and in both cohorts, the female/male ratio was 25%/75%. At the time of hospitalization, 70% of the subjects were DENV IgG seropositive, suggesting that the cohorts were predominantly associated with secondary or multiple DENV exposures, as previously reported [41]. From each subject, blood samples were obtained in the acute phase (defined as 1–7 days post fever onset (PFO)) and in the convalescent phase, after discharge from the hospital (in the 18 to 93 days range), with a median of 29 and 30 days for the DF and DHF cohort, respectively. Other clinical tests done in the acute phase upon admission, including platelet counts, hematocrit, AST, and ALT, are summarized in Table 1. The median platelet count of the DHF cohort was 75 × 1000/mm^3^ versus 95 × 1000/mm^3^ of the DF cohort. The median hematocrit of the DHF cohort was 43% and 41% in the DF cohort. The median AST and ALT of the DHF cohort were 159 and 93 (U/L) versus 113 and 89 (U/L) in the DF cohort.

### 3.2. Kinetics of IFN-γ Response and Memory Phenotypes Associated with DENV–Specific CD8+ T Cells

We previously described a DENV CD8 epitope “megapool” (MP) composed of 268 different CD8 T cell epitopes derived from DENV1-4 [6,28] that allows broad coverage of DENV-specific CD8 responses. Here, PBMCs isolated from blood samples of the DF or DHF cohorts were stimulated ex vivo with the DENV CD8 MP at 1 µg/mL. After 3 h, a cytokine capture assay and flow cytometry analysis were used to isolate IFN-γ+ and IFN-γ− CD8 T cells, as shown in Figure 1a for a representative example. No significant difference was noted in terms of % of IFN-γ-producing CD8+ T-cells as a function of DF vs. DHF disease severity (Figure 1b; Median ± IQR: DF= 0.495 ± 1.978; DHF = 0.405 ± 3.2, *p* = 0.8404 by Mann–Whitney test). Consistent with other reports [42], the CD8+ T cell response was low or undetectable in early stages (days 1–3 PFO; median ± IQR = 0.12 ± 0.24), and remained fairly stable thereafter, up to 100 days after viral infection (median ± IQR: 4–7 days PFO = 0.67 ± 4.53; 8–35 days PFO = 0.555 ± 1.778; 36–100 days PFO = 0.76 ± 1.97) (Figure 1c).

The kinetics of IFN-γ responses in CD8+ T cells are remarkably distinct from those previously reported for CD4+ T cells [17], which peaked during the first week of PFO, and decreased thereafter. Our previously published data for CD4+ T cell IFN-γ production [17] were replotted here for reference purposes in Figure 1d. We next determined the memory phenotypes of CD8+ T cells, defined by the expression of the CD45RA and CCR7 cell surface markers. No difference in the memory phenotypes of the DF vs. DHF cohorts was observed in either the total CD8+ T cell compartment or the IFN-γ+ producing CD8+ T cells subset (Figure 1e). In conclusion, CD8+ IFN-γ+ T cells were detectable from 4 days after PFO onwards and remained at steady levels over a 3–4 month PFO period. No differences were observed as a function of disease severity in the magnitude of IFN-γ+ CD8+ T cells responses and their memory phenotypes.

### 3.3. PCA Analysis Highlights Main Sources of Transcriptional Differences in the Study Cohort

**Figure 2:**
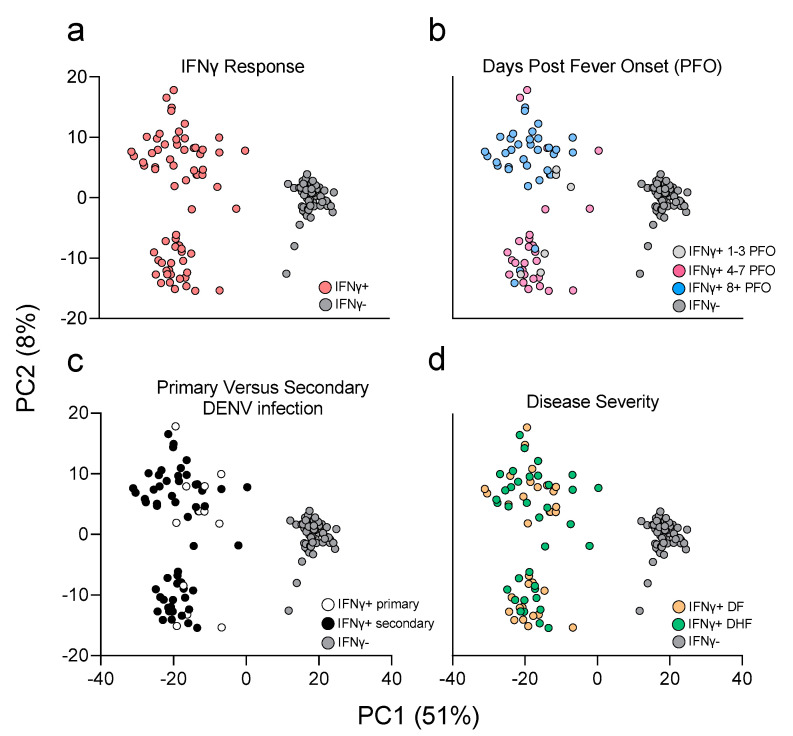
Principal component (PC) analysis of microscaled RNAseq data of DENV-specific IFN-γ+ and IFNγ− CD8+ T cells. PC1 shows separation for IFNγ− (grey) and IFNγ + (red) samples (**a**). PC2 shows separation as function of days post fever onset between 4–7 days (magenta) and 8+ (light blue), no clear separation observed for 1–3 days (light grey) (**b**). No clear pattern observed as function of primary (white) or secondary (black) DENV infection (**c**). No separation observed as function of disease severity (**d**); DF are shown in orange and DHF in green. Percentage variance explained is shown for PC1 (51%) and PC2 (8%), respectively.

The results above show that DENV-specific CD8+ IFN-γ+ T cells responses in the DF and DHF cohorts have similar magnitude and kinetics. Next, we investigated potential qualitative differences between the transcriptional profiles of the different T cell populations. To this end, total mRNA was extracted and quantified. We performed micro-scaled RNA-seq assays on the CD8+ IFN-γ+ and CD8+ IFN-γ− T cell populations, sorted after DENV CD8 MP stimulation, as described above and in previous studies [31,43]. The resulting data was subject to PCA analysis (Figure 2). As expected, a clear separation was observed between IFN-γ+ and IFN-γ− samples along the principal component (PC)1, the main source of variance (PC1 variance=51%; Figure 2a). Two distinct cluster of samples emerged on the PC2 based on the days PFO, which mapped to a first cluster encompassing early (4–7 days PFO) time points, well resolved from a second cluster encompassing later time points (8 days PFO or longer) (PC2 variance=8%; Figure 2b). No clear clustering pattern was observed in samples collected 1–3 days PFO. This is consistent with the kinetics data from Figure 1, which showed that in this time period the IFN-γ response is still developing and of low magnitude. 

Accordingly, timepoints in the 1–3 days range were excluded from subsequent analyses. Additionally, no clear separation emerged based on having a primary or secondary DENV infection, although the number of primary infections were 30% or less in both co-horts so due to the low number we have not considered this parameter in subsequent analyses (Figure 2c). Finally, no clear separation in the PCA graph was noted between DF and DHF samples (Figure 2d). In conclusion, these data suggest that while both anti-gen stimulation and time from infection are associated with clear transcriptomic differences, disease severity is not associated with prominently different transcriptomic profiles.

### 3.4. Characterization of the Transcriptional Response of DENV-Specific CD8+ T Cells following Cognate Antigen Stimulation

**Figure 3:**
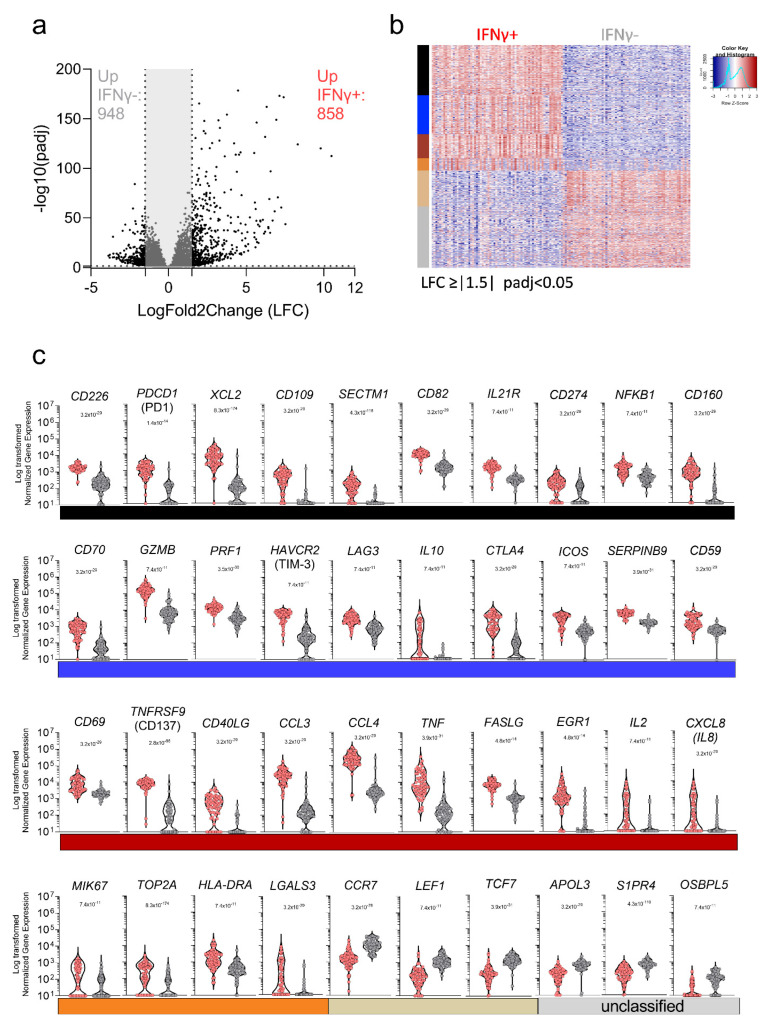
DE genes related to IFNγ signature of DENV-specific CD8+ T cells. (**a**) Volcano plot of differential expressed genes plotted based on their adjusted *p*-values and log2 fold change gene expression. (**b**) Heatmap of the most significantly differentially expressed genes obtained after comparison of IFNγ− and IFNγ+ cells and grouped based on WGCNA analysis. (**c**) Normalized counts of selected genes differentially expressed and related to the WGCNA module/function. All the analyses have been carried out considering significant genes with a log2 fold change gene expression less than −1.5 and greater than 1.5 and adjusted *p*-values less than 0.05.

Based on the PCA analysis shown above, we explored the gene signatures associated with IFNγ production from DENV-specific CD8+ T cells. When comparing IFN-γ+ CD8+ T cells and IFNγ− CD8+ T cells, a total of 1806 DE genes were identified, based on the log2 fold change (LFC) > 1.5 or <−1.5 and with *p* adjusted value (padj) < 0.05, and filtering out low-expressed genes using as the threshold of exclusion, the median expression level ≥ 10 tpm across all samples analyzed (IFNγ−/+) (Figure 3a and Supplementary Materials, Table S1). Next, we investigated the function associated with the DE expressed genes by performing a weighted gene co-expression network analysis (WGCNA) to group genes that have similar expression patterns and identified five main modules (black, blue, orange, tan, and coral). Genes that in the network construction did not belong to any specific modules were assigned to a “grey” module. The overall gene expression patterns are shown as a heatmap in Figure 3b, ordered by module color (the significant modules and the grouping by color is specified in Supplementary Materials, Table S1). The normalized counts observed for the selected genes described above are also visualized in Figure 3c. The black module included genes related to leukocyte activation and NFKB1 signaling, with a specific signature for CTL activation underlined by the joint upregulation of CD226, CD274 (PD-L1), CD160 and PDCD1 (encoding the PD1 protein) [29]. The latter was previously associated with DENV-specific CD8+ T cells with high cytotoxic potential rather than an exhaustion phenotype [44] (Figure 3c). The blue module corresponded to additional genes with cytotoxic function, including CD70, SERPINB9, GZMB, and PRF1. The blue module also included genes generally related to regulatory functions, such as HAVCR2 (encoding TIM-3), LAG3, and CTLA4 (Figure 3c). The coral module is specifically related to the IFNγ pathway and, more in general, to cytokine signaling, as shown by the co-expression of CD40LG, CCL3, TNFA, IL-2, and CXCL8 (encoding IL-8) (Figure 3c). The orange (Peru) module is specifically related to T cell activation in line with the expression of MKI67 (encoding KI-67), generally used as a marker of cell activation as well as HLA-DRA, TOP2A, and LGALS3 (encoding gal3), all related to T cell regulation and activation upon stimulation. The tan module included genes classically related to T cell differentiation, such as CCR7, LEF1, and TCF7, all associated with T cell migration and all downregulated in the IFN-γ+ CD8+ T cells, which is indicative of a more differentiated phenotype (Figure 3c). Finally, the unclustered genes, shown in grey, highlight genes related to cell metabolism and particularly to the lipid metabolism, such as APOL3, S1PR4, and OSBPL5. The analyses related to the gene function in each module are summarized in Supplementary Materials, Figure S1. In all cases, strong significance was noted for the comparisons, with *p*-values ranging from 7.4 × 10^−11^ to 8.3 × 10^−174^.

### 3.5. Gene Signature Differences in DENV-Specific CD8+ IFNγ+ Response in Early vs. Late Phase Samples and as a Function of Disease Severity 

**Figure 4:**
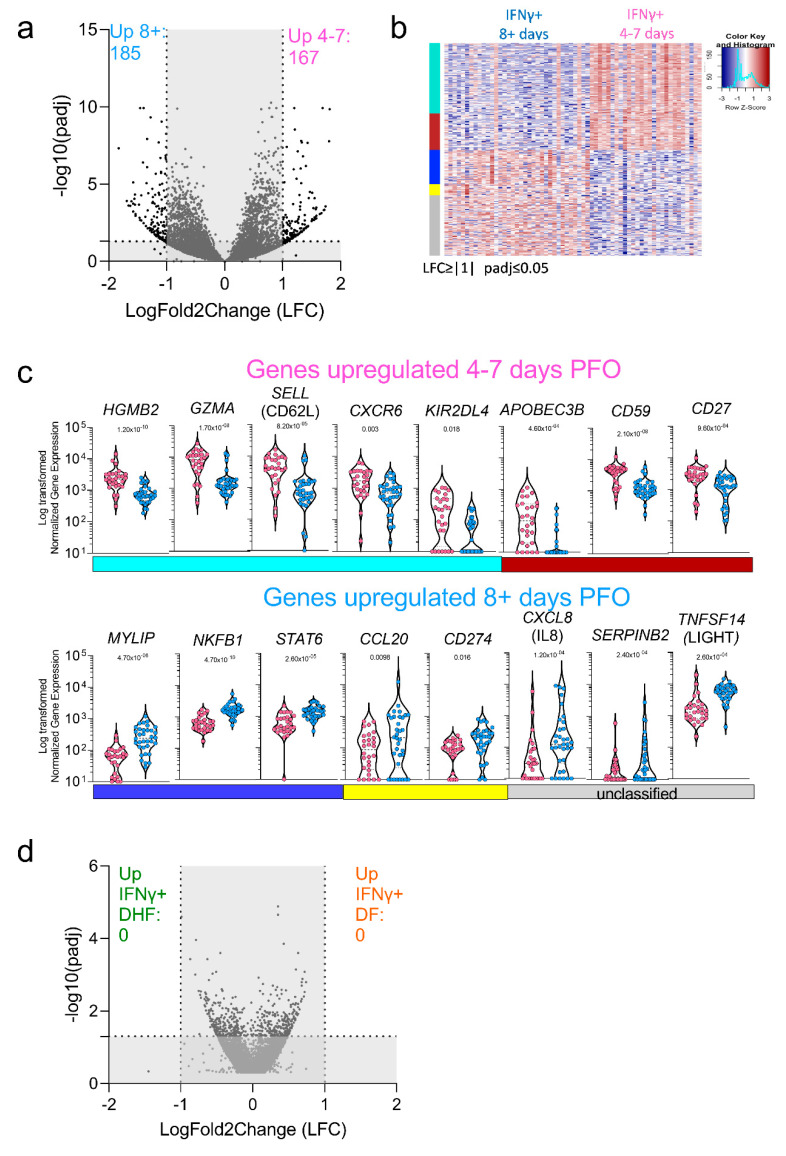
DE genes related to DENV-specific CD8+ IFNγ+ T cells signature. (**a**) Volcano plot of differential expressed genes plotted based on their adjusted *p*-values and log2 fold change gene expression in early and late phase of infection. (**b**) Heatmap of the most significantly differentially expressed genes in early (4–7 days PFO) and late (8 + days PFO) samples. (**c**) Normalized counts of selected genes upregulated in early or late samples. (**d**) Volcano plot of differential expressed genes plotted based on their adjusted *p*-values and log2 fold change gene expression in DF and DHF samples. All the analyses have been carried out considering significant genes with a log2 fold change gene expression less than −1 and greater than 1 and adjusted *p*-values less than 0.05.

In the next series of experiments, we explored the signatures associated with the DENV-specific CD8+ IFNγ+ response in early (4–7 days PFO) versus late (8+ days PFO) samples, reflective of acute and convalescent phase samples (Figure 2b). An LFC cut-off greater than 1 and less than −1 was applied in this case, with a more stringent cut-off applied for this comparison to highlight only the most significant genes and the padj < 0.05. The data are visualized in a volcano plot (Figure 4a) and in the heat map shown in Figure 4b. A total of 352 genes were differentially expressed, with 167 genes upregulated in the early response and 185 genes upregulated in the late response (Figure 4a,b). The WGCNA analysis identified four main modules with group genes that have similar expression patterns (turquoise, coral, blue, and yellow), while the unclassified genes were assigned again to a “grey” module. Figure 4b shows the heatmap for this comparison as well as the module color, while the significant modules and their function are detailed in Supplementary Materials, Table S2 and Figure S2.

The actual normalized counts observed for those selected genes are also visualized in Figure 4c. Overall p adjusted values ranged from 0.018 to 4.70 × 10^−10^. The genes upregulated in the early phase were mostly related to proliferation/cell cycle and HLA class I pathway related such as HGMB2, CCNB2, KIF15, TOP2A, APOBEC3B and genes related to the CD8 cytotoxic function such as GZMA or cell migration and adhesion such as SELL (encoding for CD62L) (Figure 4C). Conversely, genes upregulated in the late phase were not associated with cell proliferation but rather tended to be associated with chemokines pathway such as CXCL5 and CXCL8 (encoding for IL8 with a role in angio-genesis) [45] and protein metabolism such as MYLIP (encoding for IDOL involved in lipid metabolism) [46] and SERPINB9 characteristic of a more quiescent cell cycle [47] (Figure 4c). Finally, we investigated whether any differential gene signature could be discerned comparing the DF and DHF samples. As described above, DE genes were de-fined by LFC greater than 1.5 and padj < 0.05 and the volcano plot is shown in Figure 4d. Consistent with the exploratory PCA analysis shown in Figure 2c, no genes were found to be differentially expressed. In conclusion, these data show that a differential gene expression profile is associated with the early vs late phase of the response, while the transcriptomic profile associated with DENV-specific CD8+ T cells in DF versus DHF samples is remarkably similar.

## 4. Discussion

Herein we reported that specific transcriptomic profiles associated with DENV-specific CD8+ T cells, and in the acute vs recently convalescent phase. We further showed the transcriptomic profiles associated with different disease severities (DF vs DHF) are remarkably similar.

In previous studies, we characterized DENV-specific CD4+ and CD8+ T cells responses in the general population of endemic areas [6,7,17,43,48,49]. The results enabled the generation of pools of experimentally defined epitopes with broad HLA coverage spectrum and suited to analyze antigen specific T cell responses in small-volume samples [30]. The current results further exemplify how the MP approach is well suited to obtain granular characterization of antigen specific T cell responses. 

These results shown are significant in several different respects. First, they define the signature associated with DENV-specific CD8+ T cells following actual activation with cognate antigen. The transcriptomic patterns associated with DENV-specific CD8+ T cells revealed three different gene signatures. The first signature, characteristic of the acute disease stage and associated with T cell proliferation and HLA-specific activation; the second signature characteristic of transition to memory phenotype and observed in the post febrile and early convalescent stage; and a third stable signature observed in both acute and convalescent phases, related to sustained cytokines release and T cell activation. Thus, the present study details the patterns of antigen specific CD8+ T cell gene activation associated with acute DENV disease to an unprecedented level of granularity.

Our study defines, as mentioned above, the patterns of gene expression of DENV specific T cells. This is important, to rule out non-antigen specific effects associated with bystander activation. In this context, Chandele and co-authors measured T cell activation in a Thailand cohort and showed an increase of HLA-DR and CD38 markers in the DHF/DSS population in absence of an equivalent increase of IFNγ [50]. This result is consistent with a bystander activation effect driven by the pro-inflammatory environment characteristic of the severe dengue disease. The study of Waickman et al., of healthy individuals after TAK-003 vaccination, another dengue vaccine based on delivery of the envelope (Env) protein derived from DENV1-4 serotypes in a DENV2 back-bone, indeed showed that HLA-DR and CD38 markers also identified a large amount of TCR clones unrelated to Dengue-specific specificity [51].

Our study is complementary to the recent study of DENV-specific CD8+ T cells restricted to three specific HLA molecules (HLA-B*58:01, HLA-A*01:01, and HLA*24:02) [16] identified by tetramer staining, where the cells were characterized in the absence of cognate antigen stimulation. Chng et al. found two major populations in convalescent T cells characterized by the expression of CD57 and CD127 markers stable up to one year post dengue infection. Here, while we did not observe a cluster of cell populations in our convalescent samples, we did observe other markers consistent with memory differentiation, such as downregulation of TCF7, CCR7, and LEF1. Additionally, consistent with Chng et al., we detected upregulation of classical activation genes MKI-67, HLA-DR, and CD38 genes as well as CD69, ICOS, and PDCD1 (encoding PD1). Taken together, these results underline how different approaches (tetramer based and DENV-epitope stimulation) generate complementary insights into the process of activation of DENV-specific T cells.

The present analysis is consistent with the activated gene signature we previously reported for DENV-specific CD8+ T cells collected from blood bank healthy DENV- se-ropositive donors, and previous studies in acute samples by Chandele et al. [43,50]. In particular, our previous study identified genes DE in DENV specific Tem vs Temra CD8+ subsets utilizing PBMC samples from the Colombo (Sri Lanka) blood bank, which are therefore akin to the convalescent samples studied herein [43]. Despite the differences in experimental design (the Tian et al., 2019 [17] study was designed to study DE genes in different memory subsets), several genes that were identified in that study were also identified in the current analysis, including CCL3, FOSL1, TNF, XCL1, and CD160, as shown in Supplementary Materials, Table S1. It is worth noting that in these analyses we also found expression of TNFSF9 (encoding CD137/41BB) and CD69 markers, highlighting the potentiality to use the combination of these markers to identify DENV-specific CD8+ T cells and their functionality, as recently reported in the context of SARS-CoV-2 [52]. 

Similar observations were made at the level of DENV-specific CD4+ T cells, where the acute phase of infection was associated with a subset of CD4+ T cells that co-produced IL-10 and IFN-γ, but no altered phenotype was associated with DHFs [17]. Importantly, the analysis of DENV-specific CD4+ T cells in endemic areas of healthy sub-jects and severe patients also did not shown different or altered phenotypes as correlating with disease severity [17,53].

Both DENV-specific CD4+ and CD8+ T cells during acute phase of the disease ex-pressed multiple activation and inhibitory genes such as CTLA4, ICOS, LAG-3, HAVCR2 (encoding TIM-3) and IL-10 as well as effector molecules GZMB (encodes Granzyme B), and CCL4, suggesting that cytotoxic activity is exerted by both populations during acute phase of infection [29,53,54]. 

In this study, we observed a difference in the kinetics observed for the DENV-specific T cells, with a decrease in DENV-specific CD4+ T cells right after acute phase of infection (4–7 days post fever onset, PFO) and a more stable DENV-specific CD8+ T cells up to 100 days PFO. This difference in kinetics is also shown in the Chng study, highlighting the importance of DENV-specific CD8+ T cells in long-term protection [16]. 

Of particular relevance to our understanding of DENV protective immunity and DENV-associated immunopathology, we found no association with either quantitative or qualitive differences of the DENV-specific CD8+ T cell responses with disease severity. Taken together with previous studies [23,55,56], it is possible to speculate that the overall DENV-specific T cell responses play an important role in viral clearance [23], while the increased pro-inflammatory environment, bystander activation and dysfunctional innate immune responses maybe associated with immunopathology [57]. One of the limitations of this study is that peripheral CD8+ T cell responses were evaluated and may not represent tissue-specific responses. Thus, it is still the possibility that T cells could contribute to immune-mediated pathology when these cells interact with infected tissues\cells, although currently no data are available in supporting this possibility.

Indeed, previous studies have shown that dengue-infected monocytes signatures differ as a function of disease severity [58]. It is interesting to speculate about the relevance of this data for the development of DENV vaccines. The CYD TDV vaccine has been shown to be safe for subjects previously exposed to one or multiple DENV serotypes [59]. It is likely that this effect is related to antibody neutralization, and accordingly our data shows no difference between the profile of primary and secondary infections. Nevertheless, our data identifies specific gene expression pattern associated with T cells induced by natural infection and presumably contributing to protection. In this respect our data may provide a useful benchmark against which vaccine-induced responses can be compared. 

## 5. Conclusions

The lack of qualitative differences between DENV-specific CD8+ and CD4+ T cells as a function of disease severity does not support a pathogenic role of T cells in the severity of dengue infection.

## Figures and Tables

**Table 1:** General characteristics of hospitalized cohort with dengue disease.

	Dengue Hemorrhagic Fever (DHF; *n* = 20)	Dengue Fever (DF; *n* = 20)
Age (years)	14–56 [Median = 33, IQR = 19]	17–68 [Median = 30, IQR = 18.5]
Gender		
Male (%)	75% (15/20)	75% (15/20)
Female (%)	25% (5/20)	25% (5/20)
Sample Collection (years)	2014–2016	2014–2016
Days Post Fever Onset (PFO)		
Acute phase	1–7 [Median = 5, IQR= 1]	1–7 [Median = 4, IQR = 17]
Convalescent phase	18–66 [Median = 29, IQR = 1]	18–93 [Median = 30, IQR = 12]
DENV PCR positivity (Acute phase)	25% (5/20)	20% (4/20)
Antibody test positivity (Acute phase)		
IgM (%)	80% (16/20)	75% (15/20)
IgG (%)	70% (14/20)	90% (18/20)
Other lab tests in acute phase		
Platelet count (×1000/mm^3^)	13–170 [Median = 75, IQR= 106]	16–170 [Median = 95, IQR = 77]
Hematocrit (%)	32–56 [Median = 43, IQR = 10]	35–54 [Median= 41, IQR = 6]
AST (U/L)	32–697 [Median = 159, IQR = 330]	34–1507 [Median = 113, IQR = 164]
ALT (U/L)	25–1100 [Median = 93, IQR = 278]	29–484 [Median = 89, IQR = 171]

## Data Availability

The sequences used in this article have been submitted to the Gene Expression Omnibus under accession number GSE174482 (http://www.ncbi.nlm.nih.gov/geo/, accessed on 6 April 2022).

## Supplementary Materials

The following supporting information can be downloaded at: https://www.mdpi.com/article/10.3390/vaccines10040612/s1, Figure S1: Functional annotation of WGCNA modules based on DENV IFNγ+ versus DENV IFNγ− comparison; Figure S2: Functional annotation of WGCNA modules based on DENV IFNγ+ samples collected 4–7 days versus 8 or more days post-infection; Table S1: DE genes IFNγ− Versus IFNγ+; Table S2: DE genes IFNγ+ 4–7 versus 8+ days post-fever onset (PFO).
