# Supplementary figures and images for: Transcriptomics of Acute DENV-Specific CD8+ T Cells Does Not Support Qualitative Differences as Drivers of Disease Severity
Source: Vaccines (Basel). 2022 Apr 14;10(4):612. doi: 10.3390/vaccines10040612 (PMC9029181; doi:10.3390/vaccines10040612)

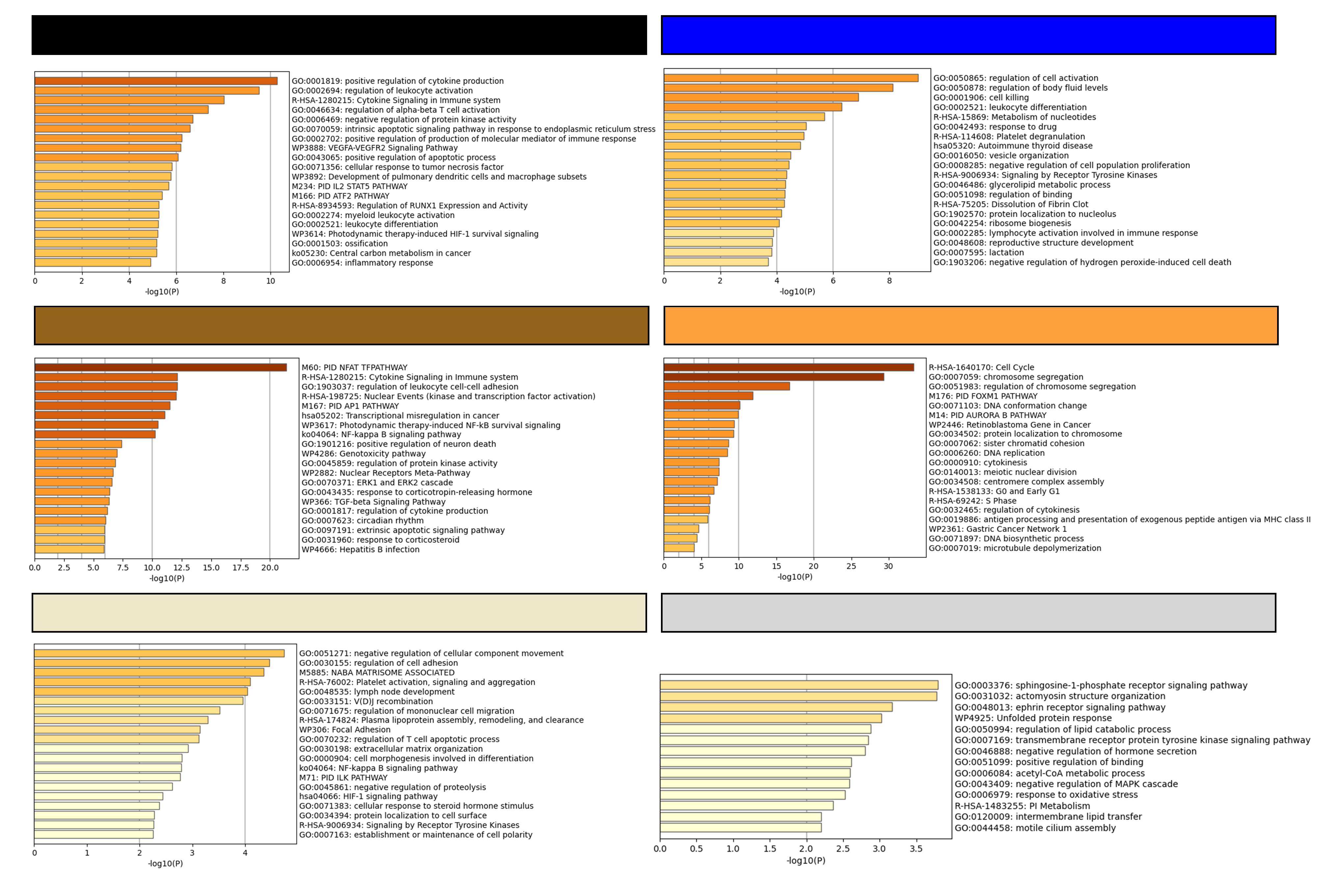

Supplement: Supplementary file 1 [file vaccines-10-00612-s001.zip › Figure S1.tif]

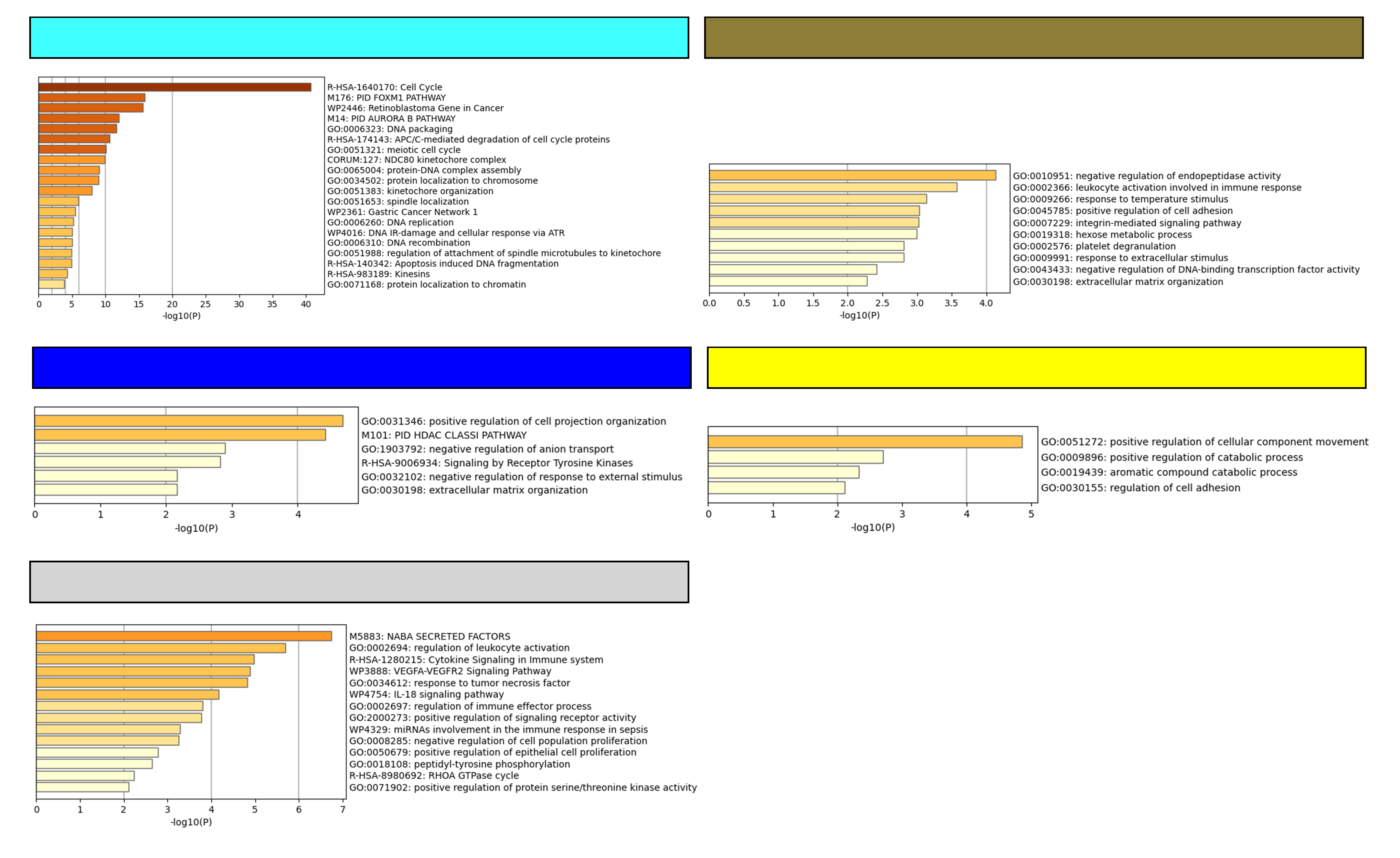

Supplement: Supplementary file 1 [file vaccines-10-00612-s001.zip › Figure S2.tif]
